# Potential value of mitochondrial regulatory pathways in the clinical application of clear cell renal cell carcinoma: a machine learning-based study

**DOI:** 10.1007/s00432-023-05393-8

**Published:** 2023-09-25

**Authors:** Xiaochen Qi, Yangyang Ge, Ao Yang, Yuanxin Liu, Qifei Wang, Guangzhen Wu

**Affiliations:** 1 Department of Urology, The First Affiliated Hospital of Dalian Medical University, Dalian, 116011 China; 2 Department of Anesthesiology, The First Affiliated Hospital of Dalian Medical University, Dalian, 116011 China

**Keywords:** Clear cell renal cell carcinoma, Mitochondria, Machine learning, Bioinformatics, Immunotherapy, Target therapy, Prognostic model

## Abstract

**Background:**

Renal clear cell carcinoma (RCC) is a common cancer in urinary system with increasing incidence. At present, targeted therapy and immunotherapy are the main therapeutic programs in clinical therapy. To develop novel drugs and provide new ideas for clinical therapy, the identification of potential ccRCC subtypes and potential target genes or pathways has become a current research focus.

**Aim:**

The aim of this study was to explore the underlying mechanisms of mitochondrial function in ccRCC. This regulatory pathway is closely related to tumor development and metastasis in ccRCC patients, and their abnormal changes may affect the prognosis of cancer patients. Therefore, we decided to construct a prognostic model of ccRCC patients based on mitochondrial regulatory genes, aiming to provide new methods and ideas for clinical therapy.

**Result:**

The 5-year survival prediction model based on iterative LASSO reached 0.746, and the cox model based on coxph reached C-index = 0.77, integrated c/D AUC = 0.61, and integrated brier score = 0.14. The rsf model based on randomForestSRC was built with C-index = 0.82, integrated c/D AUC = 0.69, and integrated brier score = 0.11. The results show that mitochondrial regulatory pathway is a potential target pathway for clinical therapy of ccRCC, which can provide guidelines for clinical targeted therapy, immunotherapy and other first-line therapy.

**Supplementary Information:**

The online version contains supplementary material available at 10.1007/s00432-023-05393-8.

## Introduction

Renal cell carcinoma, as one of the most common tumors of the urinary system, accounts for 3% of all cancers, with the highest incidence in Western countries. In general, the incidence has increased by about 2% per year globally and in Europe. Clear cell renal cell carcinoma is the most common solid lesion in the kidney, accounting for about 90% of all renal malignancies. It includes different RCC subtypes with specific histopathological and genetic characteristics. The male to female ratio is 1.5: 1. The average age of patients with the disease tends to be younger (Sung et al. 2021). Several proven risk factors have been identified, including smoking, obesity and high blood pressure. These are considered clear risk factors for RCC (Cairns 2010). Clear cell renal cell carcinoma is the largest pathological subtype of renal carcinoma, accounting for more than 75%. It is usually found during surgery that the tumor incision surface is golden yellow, often accompanied by bleeding and necrosis (Cohen and McGovern 2005). Loss of the 3p chromosome and mutation of the von Hippel-Lindau (VHL) gene on the 3p25 chromosome are often found. The loss of VHL protein function contributes to the occurrence, progression and metastasis of tumors. The 3p locus contains at least four additional ccRCC tumor suppressor genes (UTX, JARID1C, SETD2, PBRM1) (Gossage et al. 2015; Thompson et al. 2018). ccRCC generally has a poorer prognosis than other classifications, but this difference disappears after adjustment for stage and grade. Therefore, ccRCC itself is heterogeneous and has a different prognosis (Jonasch et al. 2021). Based on this idea, clinicians continue to identify potential subtypes of renal cancer and develop its potential ability to guide prognosis and clinical therapy.

In addition to being the key organelles of energy generation in the cell, mitochondria also participate in the metabolic processes such as apoptosis, free radical production and lipid metabolism. Several studies have reported that abnormal mitochondrial function contributes to the pathology of many common diseases, including neurodegeneration, metabolic diseases, heart failure, ischemia–reperfusion injury, infections in protozoa and cancer (Annesley and Fisher 2019). Mitochondria are, therefore, an important drug target for these highly prevalent diseases. Several strategies aimed at therapeutically restoring mitochondrial function are emerging, and a handful of drugs have entered clinical trials. Mitochondria are maternally inherited and originated as organelles of symbiotic bacteria. They co-evolved with the host, so most mitochondrial proteins are nuclear encoded. However, mitochondria retain a small 16 kb DNA genome that encodes tRNAs, rRNA, and proteins essential for respiration. Cells have hundreds of mitochondria and can be wild-type or a mixture of wild-type and mutant types, a state known as heterogeneity. Mitochondria are important bioenergy and biosynthesis factories that are essential for normal cell function and human health (Nunnari and Suomalainen 2012). Otto Warburg proposed that mitochondrial respiratory defects were a potential basis for aerobic glycolysis and cancer, known as the Warburg effect (Vaupel et al. 2019). However, in fact, the Warburg effect can only be used as the basis for FDG-PET tumor imaging, and not all tumors have this aerobic glycolytic property (Czernin et al. 2013). Mitochondrial respiratory defects are not usually the cause of aerobic glycolysis, nor are they usually selected for during tumor evolution. In most cancers, it is carcinogenic driver mutations such as activation of K-ras, c-Myc, and phosphatidylinositol-3 (PI3) kinases or loss of phosphatase and tensin homologues and p53 that promote glycolysis, rather than mutations in the inactivated mitochondrial respiratory complex. Most cancers always preserve mitochondrial function, including respiration. Some tumors have high levels of oxidative phosphorylation, while others still retain mitochondrial respiration and other functions. Quantified by flux analysis in cultured cells, it was found that AKT conversion did not significantly affect respiration, while Ras conversion reduced respiration, but most ATP was still produced by oxidative phosphorylation. Functional tests of mitochondrial activity requirements in cancer have revealed their importance. The inactivation of the mitochondrial transcription factor Tfam depletes the mitochondria in tumor cells, thus impairing the growth of K-ras lung tumors. Depleting the mtDNA of tumor cells by poisoning mtDNA replication to produce r0 cells can significantly disrupt tumor development. In addition, selection for recovery of MTDNA-depleted r0 tumor growth was associated with horizontal transfer of the mitochondrial genome in host tissue and respiratory recovery (Kroemer and Pouyssegur 2008; Wallace 2012; Klein et al. 2020; Missiroli et al. 2020). These and other findings suggest that the role of mitochondria in cancer is not as simple as Warburg thought. Instead, they point to the importance of mitochondrial function for tumor growth. Therefore, we decided to identify potential subtypes of ccRCC and construct prognostic models based on the expression levels of regulatory genes of mitochondrial composition and function, and reveal the guiding significance of mitochondrial regulatory mechanisms for the clinical therapy of ccRCC.

## Material and methods

### Data resource

RNA-seq and clinical information of ccRCC/KIRC samples were obtained from The Cancer Genome Atlas (TCGA) database by TCGAbiolinks R package (tumor *n* = 537, control *n* = 72) (Tomczak et al. 2015; Colaprico et al. 2016). In addition to the sample information of ccRCC/KIRC patients, cancer types of 32 different organs and tissues were also obtained for pan-cancer analysis. Gene expression information and resistance data of cell lines were obtained from the Genomics of Drug Sensitivity in Cancer (GDSC) database to predict resistance in ccRCC samples(Yang et al. 2012).

### Gene set acquisition and potential subtype identification

The mitochondrial regulatory gene set was obtained from 1136 human mitochondria-related genes included in the MitoCarta3.0 database (Rath et al. 2021). Based on the unsupervised consensus clustering algorithm and the step analysis of Boruta algorithm, positive and negative related genes were divided and selected, and the sample subtype identification was completed. GO and KEGG databases were used for pathway enrichment analysis of the screened genes to determine whether the function of the screened genes was related to mitochondria (Kanehisa et al. 2017; The Gene Ontology Consortium 2019). All ccRCC samples were identified as clusterA (High-Mitopathway enrichment) and clusterB (Low-mitopathway enrichment) groups.

### Immunoinfiltration analysis of subtypes

Tumor infiltrating immune cells are invasive immune cells isolated from tumor tissue. A successful anti-tumor immune response requires the presence, activation, and co-stimulation of all lymphoid components of the immune system, including different populations of T cells, B cells, dendritic cells, natural killer cells (NK cells), bone marine-derived suppressor cells (MDSC), neutrophils, and macrophages. The process of malignant tumor is not only the accumulation of tumor cells, but also the formation of microenvironment by endothelial cells, fibroblasts and infiltrating immune cells. Cells in the tumor microenvironment play an important role in tumor development, invasion and metastasis. These cells influence tumor diagnosis, survival outcome, and sensitivity to clinical immunotherapy. Seven immune infiltration quantization algorithms including TIMER, CIBERSORT, Cibersort-ABS, QUANTISEQ, MCPCOUNTER, XCELL and EPIC were used to calculate the immune cell infiltration state of ccRCC samples, and heat maps were drawn to compare the differences in the degree of immune cell infiltration of the two subtypes (Newman et al. 2015; Becht et al. 2016; Li et al. 2017; Aran et al. 2017; Finotello et al. 2019; Racle and Gfeller 2020). The proportion of immune cells and stromal cells in a tumor has a significant impact on prognosis, and immune cells and stromal cells are two major types of non-tumor components in the tumor microenvironment, and have been shown to be of great value for tumor diagnosis and prognostic evaluation. The immune score and stromal score calculated based on estimate algorithm are helpful for the quantification of immune and stromal components in tumors. Based on estimate algorithm, we can obtain three scores: (1) stromal score (describing the matrix in the tumor tissue) (2) immune score (describing the infiltration of immune cells in the tumor tissue) (3) tumor purity (describing the tumor purity). Based on DNA methylation site markers, we calculated the methylation score MeTIL of tumor-infiltrated lymphocytes to evaluate the degree of tumor immune invasion from the perspective of DNA methylation (Lu et al. 2021). To further determine the degree of tumor response to immunotherapy, we extracted the gene expression of key sites of immunotherapy and compared whether there were differences among different subtypes.

### Drug resistance analysis

The GDSC database is currently the largest public database of information on drug sensitivity and molecular markers of cancer cells. Based on multiple studies and database information, the database is integrated and re-analyzed to describe the sensitivity and response of different tumor cells to drugs at the three search levels of cell, drug and molecule. The drug resistance analysis was mainly based on the gene expression data and drug resistance data of each cancer cell line in the GDSC database, and the model was constructed by ridge regression. After ccRCC samples were input into ridge regression prediction model, the drug resistance data of each ccRCC sample was obtained. We drew a box chart based on the resistance of ccRCC subtypes to various clinical cancer targeted therapies to compare the differences in drug resistance, and calculated the statistical differences based on wilcox-test. pRRophetic R package is used for data analysis (Geeleher et al. 2014).

### Prediction model construction

LASSO-COX regression has been used to construct polygenic clinical prognostic models. The purpose of this algorithm is to reduce the variables included in the final prediction model and minimize the overfitting degree of the model while ensuring the accuracy of the model. However, lasso regression algorithm needs to set seeds, lasso is highly dependent on seeds when allowed, because the algorithm itself needs cross-validation, and the cross-validation process is to randomly select samples. So once we change the seed, the optimal lambda will change, and the resulting feature will change. Therefore, we decided to improve the classical LASSO regression algorithm by iterating LASSO to produce genes that are retained under consensus (Sveen et al. 2012). We ran 1000 times LASSO regression in seed-independent sequences to sequence the frequency of occurrence of the genes that were preserved. The area under ROC curve (AUC) further selects the smallest combination of genes associated with survival. Consensus is the feature that is retained at high frequency after multiple runs of lasso (independent of seeding) and can be considered to have the most impact on the topic of interest. The order of frequency represents the degree of influence of these features, and then these features are incorporated into the cox model in turn, and the inclusion stops when the AUC reaches its peak, at which time the model is optimal and contains the least features. We show the process of model construction in the form of AUC change curve, and plot the 5-year ROC curve at the peak of AUC. After riskscore (riskscore = Σgene exp × gene coef) was calculated, COX regression model and random survival forest model were incorporated into the sample combined with clinical characteristics, TNM grading, staging and other variables, and two prognostic models for ccRCC patients were constructed.

### Model performance evaluation

Survival analysis is a task that deals with event time prediction. In addition to Cox regression models, a number of survival analysis models based on machine learning algorithms have recently emerged, most of which generally lack interpretability due to their complexity. Survex (https://github.com/ModelOriented/survex), as a tool to explain survival analysis models, can explain not only Cox regression models, but also randomForestRSC models based on machine learning algorithms. Based on the survex package, we calculated a variety of time-dependent model evaluation indicators of cox model and rsf model, including C-index, C/D AUC (cumulative/dynamic), and brier score. We then analyzed the importance and bias dependence of each feature, and performed local interpretation of each feature based on the SHAP algorithm to evaluate the importance of the variable in predicting the two selected observations. Compare cox and rsf two prediction models and choose a better prediction model as the final choice.

## Results

### Clustering and gene screening

**Fig. 1:**
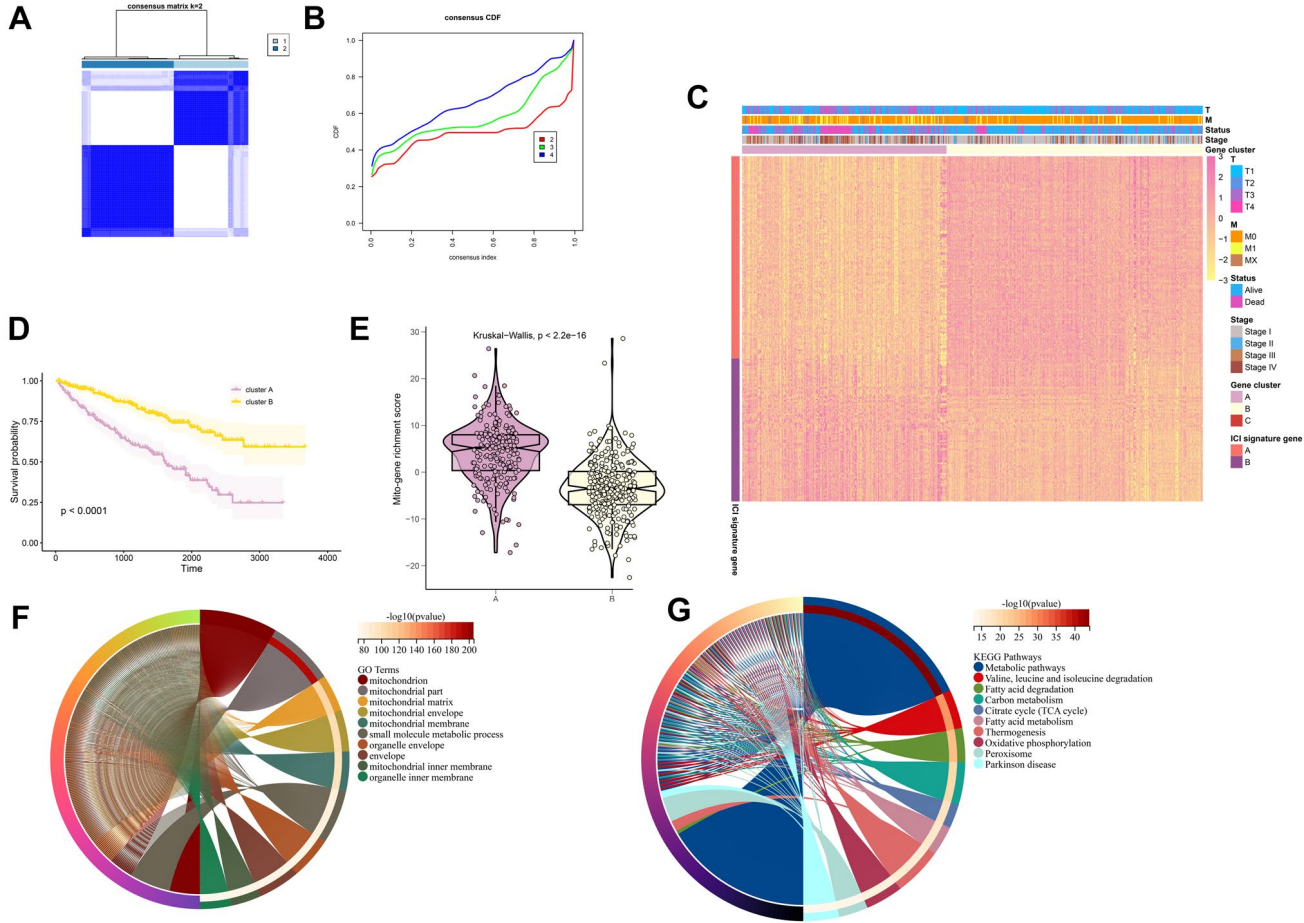
Selection of mitopathway genes and Clustering of ccRCC samples. **A** Heatmap shows the condition of samples clustering. **B** Cumulative distribution function of samples clustering. **C** Heatmap shows the gene expression after Boruta algorithm of two different clusters. The color blocks on the right indicate the distribution of gene expression and other clinical information in different groups. **D** The survival curve of the two clusters. **E** Violin plot shows the difference of the mitopathway enrichment in different clusters. **F** GO database enrichment analysis. **G** KEGG database enrichment analysis

In unsupervised cluster analysis, we tried a variety of groupings. According to the evaluation curve of CDF of clusters, we finally selected two clusters, clusterA and clusterB, and divided the positive and negative correlation sets of all genes. Then, dimension reduction of 1136 mitopathway genes (Supplementary Table 1) was carried out based on boruta algorithm, and the number of genes was reduced to 273 (Fig. 1A–C, Supplementary Tables 2–3). The survival curves drawn according to the two clusters suggest that there are significant survival differences between the two clusters (Fig. 1D, *p* < 0.001). The Mitopathway gene richment score of each sample was calculated based on PCA algorithm based on positive and negative gene sets. As can be seen from the violin chart, there are obvious differences in richment score of the two clusters. ClusterA obviously has high-mitopathway enrichment, ClusterB obviously has low-mitopathway enrichment. The *p*-value obtained by kruskal.test is 2.2e–16, which is significantly lower than 0.05 (Fig. 1E). The selected genes were input into GO and KEGG databases for analysis and their functional annotations were obtained (Fig. 1F, G). We found that these genes mainly function to regulate the composition of mitochondria, form mitochondrial membrane structure, and perform metabolic pathways dependent on mitochondria (degradation of valine, leucine and isoleucine, degradation of fatty acids, carbon metabolism, citric acid cycle (TCA cycle), and so on. Mitochondrial membrane fatty acid metabolism).

### Immune cell infiltration landscape and immune-related score

**Fig. 2:**
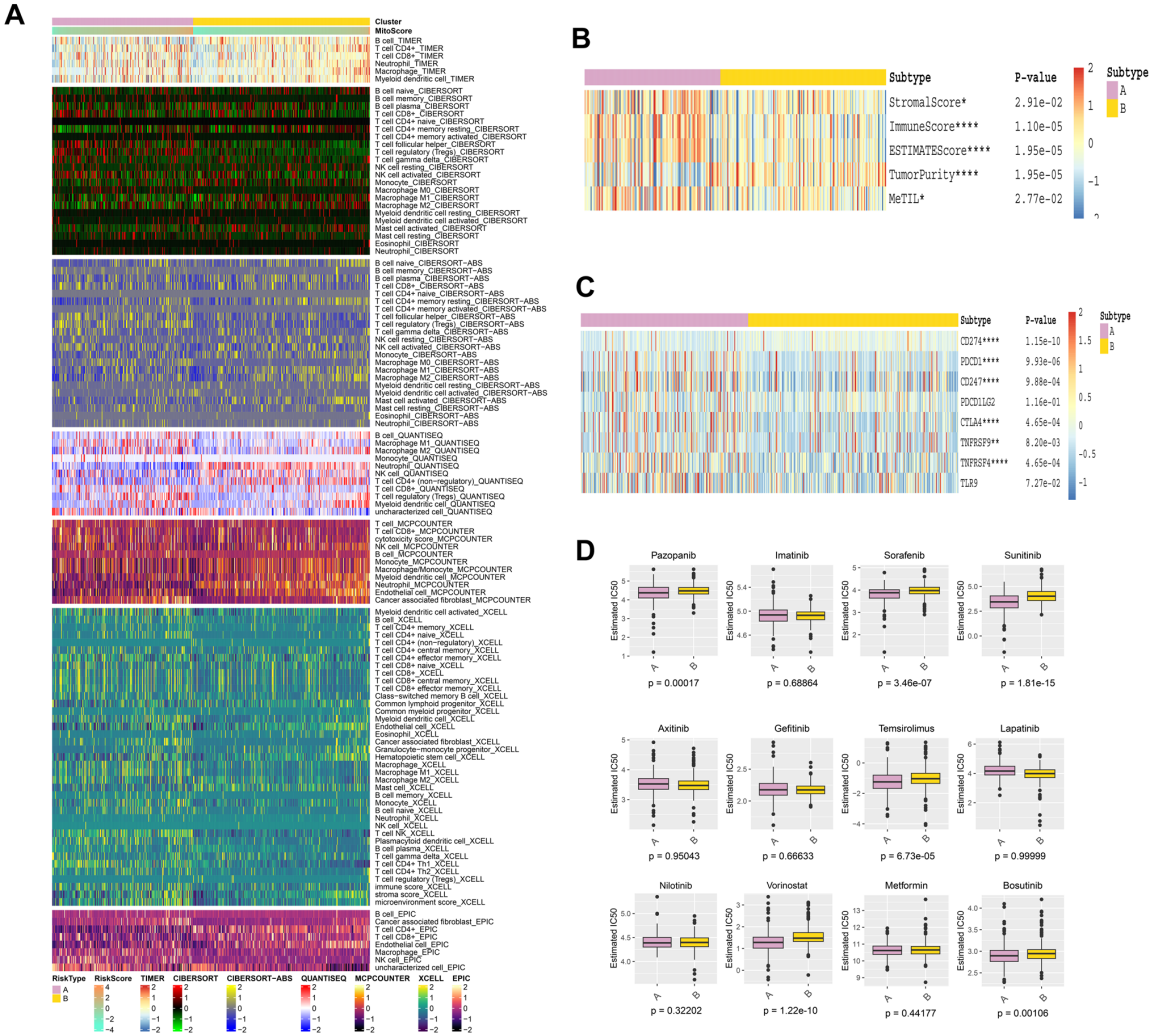
Heatmap shows the difference of immune infiltration and scores between the two clusters and the difference of drug sensitivity results of multiple targeted drugs. **A** The difference of immune landscape in different clustering algorithms. The lower color blocks represent different levels of immune cells. **B** The difference of immunization scores in different groups. The color blocks on the right represent different levels of immunity scores. **C** Differences of immune checkpoint regulatory genes in different groups. The color blocks on the right represent different levels of immunity scores. **D** The box plot shows the drug sensitivity of different clusters to 12 common clinically targeted drugs, expressed as IC50

The levels of immune components obtained by seven immune cell infiltration fitting algorithms are presented in the form of heatmap (Fig. 2A, Supplementary Table 4). The correlation between immune component levels and mitochondrial function was revealed through labeling clustering and sequencing of samples based on mitopathway score. Immune-related score calculated based on the ESTIMATE: stromal score, immune score, tumor purity, tumor-infiltrating lymphocyte methylation score (MeTIL) and Immune checkpoint related genes also suggested that there were significant differences in immune microenvironment between the two clusters (Wilcox-test *p* < 0.05, Fig. 2B, C, Supplementary Tables 5, 6). The IC50 prediction results of 12 anticancer drugs based on the GDSC database are presented in the form of box charts, and the Wilcox-test *p*-value is marked at the bottom of each box chart (Fig. 2D, Supplementary Table 7). We found that pazopanib, sunitinib and sorafenib, the three first-line ccRCC targets, had significant differences in IC50 in different clusters (*p* < 0.05), suggesting that the expression of mitopathway can guide the clinical application of these targeted drugs to some extent. Mitochondrial regulatory genes are expected to become Nova biomarkers guiding clinical therapeutic use of ccRCC.

### Construction of ccRCC prediction model

**Fig. 3:**
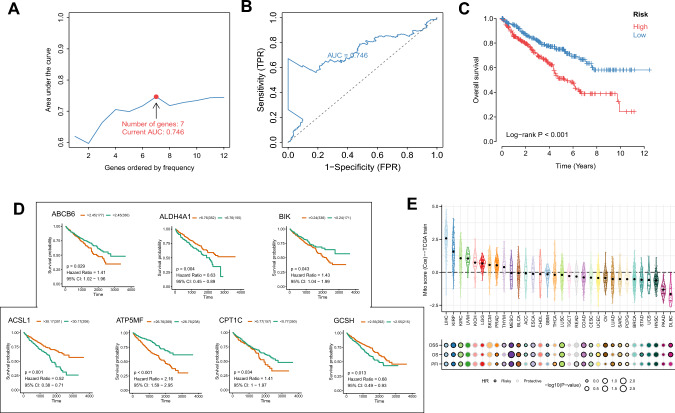
Model construction based on mitopathway genes. **A**, **B** Iterative LASSO analysis indicated that 7 genes were screened when AUC reached the maximum, and AUC_max_ = 0.746. **C** Survival curve of High/Low risk group. **D** Survival group of 7 selected genes with High/Low expression. **E** The riskscore values of pancarcinoma samples were calculated based on the expression and coefficient of the selected genes, and the riskscore was judged as a risk or a protective role

Iterative LASSO was used to further screen 273 mitochondrial regulatory genes. After 1000 iterations, 7 genes were finally selected for the construction of the model, respectively: ABCB6, ACSL1, ALDH4A1, ATP5MF, BIK, CPT1C, GCSH (Supplementary Table 8). The corresponding 5-year survival prediction AUC at this time is 0.746, and the survival curve difference p-value of the risk groups is much less than 0.001, which confirmed the effectiveness of the iterative LASSO algorithm (Fig. 3A–C). We sequenced the ccRCC samples according to these gene expression levels, and took the loci that could make the greatest difference in survival between the two groups of samples (the lowest *p*-value) as the cut-off value (Fig. 3D). The results of survival curve indicated that the expression difference of 7 genes was related to the survival difference of patients. After the riskscore of all samples was calculated (Supplementary Table 9), it was fused with clinical data to build a prediction model. Based on the expression levels of 7 genes and their coefficients, the riskscore of pan-cancer samples was calculated to provide an overview of whether the mitochondrial pathway is a protective or a risk pathway in different cancers (Fig. 3E). We found it very interesting that there are two common pathological subtypes of kidney cancer: riskscore of mitochondrial pathway plays a risk role in both clear cell renal cell carcinoma (ccRCC) and papillary renal cell carcinom (pRCC), which provides a good idea for drug development of renal cell carcinoma. We can look for pathways that act on the same organ or tissue at the same time, thereby extending the range of action of the drug. In R, cox regression model is constructed based on coxph function and rsf model is constructed based on randomForestSRC function.

### Variable interpretation and comparison of prediction models

**Fig. 4:**
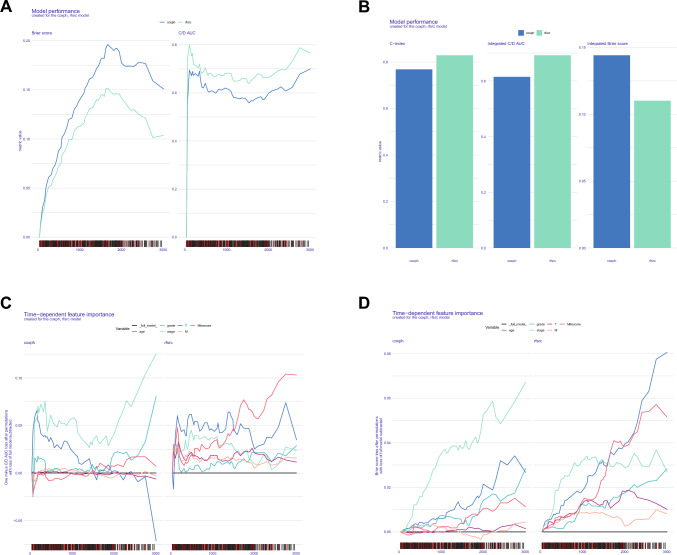
Evaluation and comparison of prediction models. **A** Time-dependent model performance. **B** Average performance of the model. **C**, **D** Changes in the importance of time-dependent feature

**Fig. 5:**
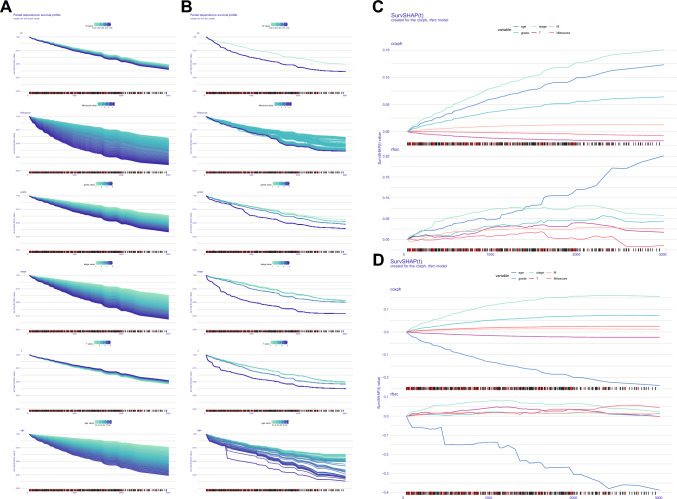
Evaluation and comparison of prediction models. **A**, **B** Partial dependence of each feature (Time dependent). **C**, **D** The SHAP value of each feature over time

Due to the complexity of machine learning algorithms, we cannot directly apply nomogram and other display methods. Therefore, we adopted survex R package to compare the two models and explain the global/local features. Through calculation and comparison of C-index, AUC and Brier score (Fig. 4A, B, Supplementary Tables 10–12), we found that the prediction performance of the rsf model was comprehensively superior to that of the cox model. After each variable was split, variable interpretation was carried out. In the global interpretation, we found that the influence of stage variable in the cox model on the model gradually increased over time (Fig. 4C, D). In rsf model, riskscore and age are the main influencing variables. In the degree of dependence curve plot, the wider the curve area, the more obvious the change of the variable can cause the fluctuation of the model (Fig. 5A, B). We find that riskscore is of great importance in both the cox model and the rsf model. In the SHAP algorithm, we found that with the increase of time, the SHAP value of riskscore in the rsf model with two observed values (12, 32) would gradually increase and occupy the dominant position of model variables(Fig. 5C, D). The comparison and interpretation results of cox model and rsf model indicate the effectiveness and accuracy of the model construction, indicating that mitochondrial regulation related genes have certain application value in the clinical survival prediction of ccRCC.

## Discussion

Renal cell carcinoma (RCC) is a kind of malignant tumor that is not sensitive to radiotherapy or chemotherapy. Currently, effective tumor therapy mainly relies on a variety of molecular targeted therapeutic drugs targeting vascular endothelial cell growth factor (VEGF), platelet-derived growth factor (PDGF) and mammalian target protein of rapamycin (mTOR) and immunotherapy targeting immune checkpoints such as PD-1 and PD-L1 (Yoon 2017; Chen et al. 2019; Braun et al. 2020; Lai et al. 2021; Qi et al. 2022). Renal cell carcinoma (RCC) is also considered to be a metabolic disease in many studies, mainly due to the presence of large amounts of carbohydrate, cholesterol, and fat metabolic reprogramming in renal cell (Wettersten et al. 2017). In normal cells, a large portion of glucose is metabolized to pyruvate through the TCA (Krebs) cycle in the mitochondria and oxidative phosphorylation, which is almost completely oxidized to CO2, resulting in a large amount of ATP (Tsvetkov et al. 2022). Pyruvate can be metabolized into lactic acid only when oxygen is restricted. Instead, most cancer cells convert most glucose into lactic acid, regardless of oxygen availability (the Warburg effect). In addition, tumor cells increase ROS production, thereby enhancing their antioxidant defenses to avoid oxidative damage and maintain ROS homeostasis. Because of this, key enzyme proteins and intermediates in the TCA cycle and oxidative phosphorylation have become potential targets for many cancer targeting drugs. Clear cell renal cell carcinoma is the most common pathological type of renal cell carcinoma. In 70–90% of patients with clear cell renal cell carcinoma, the VHL gene is inactivated, resulting in significantly increased hypoxia-inducing factor (HIF) levels in the cancer cells in the normoxic state (Zhang and Zhang 2018; Thompson et al. 2018). HIF can inhibit mitochondrial glucose oxidation by up-regulating the expression of pyruvate dehydrogenase kinase (PDK), a key protein kinase that regulates mitochondrial glucose oxidation metabolism, and then up-regulating the expression level of intracellular glycolytic enzyme. The inhibition of mitochondrial function in cancer cells in this anaerobic state can inhibit the apoptosis process of the mitochondrial pathway, reduce the levels of alpha-ketoglutarate, a circulating metabolite of tricarboxylate, and mitochondria-related ROS, and thus inhibit the function of P53. P53 has been proved to have tumor suppressor function, which can inhibit the expression of pyruvate dehydrogenase kinase 2 (PDK2), thus activating mitochondrial oxidative metabolism and promoting TCA cycle (Zhang et al. 2013; Harlander et al. 2017). In addition, p53 can induce mitochondrial GLS2 expression to enhance GSH synthesis and alpha- ketoglutarate, thereby promoting TCA cycling. P53 function is often impaired in tumors. Idasanutlin (RG7388), a small molecule that blocks the negative regulation of P53 in rat double microgene 2 (Mdm2), is currently in Phase III trials (Konopleva et al. 2020). It has been shown that RG7388 effectively reduces cell proliferation and induces p53-dependent pathways, cell cycle arrest and apoptosis, thereby inhibiting tumor growth. Meanwhile, ALRN-6924, a dual-targeted inhibitor of Mdm2/MdmX, has been tested in Phase I clinical trials (Saleh et al. 2021). The present results suggest that it stably activates p53-dependent transcription at the single-cell and single-molecule levels, and has good tolerance and antitumor activity in patients with solid tumors or lymphomas carrying wild-type TP53.

Studies have shown that OXPHOS can provide ATP for tumor proliferation. The electron transport chain (ETC) is an important component of OXPHOS, which consists of the complex I-IV, CoQ, and Cyt c and is required for tumor growth. As a major producer of proton gradients in ETC, complex I is a suitable target for the development of OXPHOS inhibitors. Early metformin and BAY87-2243 received much attention for their ability to inhibit complex I, but their low potency and severe side effects prevented their further development (Foretz et al. 2014; Mallik and Chowdhury 2018; Du et al. 2022). Petasin (PT) is a complex I inhibitor that mainly inhibits tumor growth in animal models with high efficiency and low toxicity (Heishima et al. 2021). In addition, the human epidermal growth factor receptor 2 (ERBB2) inhibitor mubritinib has anticancer effects by inhibiting complex I (Baccelli et al. 2019).

Multidrug resistance (MDR) in tumor cells is also related to mitochondria. MDR is one of the main causes of chemotherapy failure. The occurrence of MDR is associated with a variety of proteins on the cell membrane, such as the energy-dependent P-gp protein, which can expel chemotherapy drugs from the cell with the help of ATP. The ATP needed for P-gp to function comes mainly from the mitochondria. With high energy demand, mitochondria produce more ATP through glycolysis (Kopecka et al. 2020). Considering the multitude of drugs available for the clinical treatment of ccRCC, we decided to analyze the sensitivity of ccRCC samples with differential cuprotosis expression to these commonly used drugs, starting with common chemotherapy drugs and targeted therapies for kidney cancer. Therefore, all 12 drugs selected from the GDSC database are first and second-line treatments for kidney cancer. The IC50 prediction results for all drugs show significant differences between the high and low cuprotosis expression groups, with most drugs in the three groups showing a trend of increased or decreased IC50 values. It is gratifying to note that the first-line drugs in this subgroup: Sorafenib, Sunitinib, and pazopanib all show good resistance differences. This indicates that the classic ccRCC drugs used in clinical practice are related to cuprotosis. This preliminary result confirms our hypothesis from a clinical treatment perspective and suggests that the differential expression of cuprotosis still has guiding significance in the selection of currently used therapeutic drugs.

Based on these current research hotspots, we believe that mitochondria play an important role in the alteration of glucose and lipid metabolism in cancer. The key regulatory genes of mitochondria must play a key role in ccRCC, a type of cancer with obvious metabolic variation. MitoCarta3.0 which was published in 2021 is an emerging mitochondrial gene database. At present, the database contains a total of 1136 human mitochondrial pathway genes and 1140 mouse mitochondrial pathway genes, which is the most comprehensive gene bank for explaining mitochondrial function, structure and metabolism in the public database. To screen key genes broadly, we decided to include all human mitochondrial pathway genes in the initial study, rather than focusing on certain key pathways, to ensure maximum refinement of the final predictive model's ability to interpret mitochondria. The 7 mitochondrial pathway genes with large regulatory range ABCB6, ACSL1, ALDH4A1, ATP5MF, BIK, CPT1C and GCSH obtained by the final iterative LASSO screening also suggests that the final prediction model has a broad explanatory ability for mitochondrial function, rather than being limited to certain mitochondrial functional pathways. After comparison with Mitocarta database and literature review, we found that ABCB6, as a regulatory factor of ATP binding box, is mainly responsible for the transport of metal ions, cofactors and small molecules. ACSL1 is mainly responsible for regulating lipid metabolism and fatty acid oxidation balance (Quan et al. 2021), while ALDH4A1 and GCSH play important roles in amino acid metabolism, and are responsible for regulating the production and transport of proline and glycine, respectively (Lorenzo et al. 2021). ATP5MF is an important component in the regulation of oxidative phosphorylation: Complex 5 (Zhang et al. 2022). BIK is directly related to mitochondrial apoptosis (Chinnadurai et al. 2008). CPT1C is mainly responsible for carnitine transport and lipid metabolism (Fadó et al. 2023). It can be seen that almost most of the genes are related to the changes of mitochondria in cancer cells, and it can be considered that these genes have a potential regulatory relationship with the proliferation and metastasis of cancer cells. A more interesting phenomenon is that most of the genes that were screened were concentrated in cellular metabolic functions. For this phenomenon, we believe that there are several possibilities: 1.Gene regulation in cell metabolism: The mitochondrial pathway is closely related to cell metabolism. To maintain normal biological activities, cells must carry out various metabolic processes, such as energy production, organic synthesis, decomposition, and so on. Therefore, the enrichment of metabolism-related genes in the mitochondrial pathway is expected. 2. The main function of the mitochondria: The mitochondria is an organelle within the cell whose main function is to produce the energy required by the cell. Mitochondria provide energy through ATP produced during cellular respiration. ATP is involved in many metabolic pathways in the cell, including fatty acid metabolism, glycolysis, ketone body synthesis, etc. Therefore, gene functions associated with these metabolic processes may be highly enriched in the mitochondrial pathway. 3. The relationship between mitochondrial pathway and metabolic diseases: The mitochondrial pathway is closely related to the development and progression of many metabolic diseases. In these diseases, gene mutations or functional abnormalities related to energy metabolism and cellular respiratory function may lead to the occurrence of the disease. Therefore, genes that play an important role in metabolic function may receive more attention in the functional enrichment of mitochondrial pathways.

At present, the construction of prediction models based on gene screening is based on model variables such as forest map, nomogram and so on. Here, we hope to find different model display methods to interpret the model we constructed from a new perspective. At present, the large-scale development of machine learning has provided great help for the construction of clinical prediction models. In this paper, we use cox regression and random survival forest, a well-known variant of random forest in machine learning algorithms, to compare two different model construction methods, and use the survex R package to quantify the model (Taylor 2011). The results show that the machine learning random forest algorithm is ahead of the traditional cox regression algorithm in many aspects, and thanks to the help of survex, the former 's complex internal algorithm can be explained globally and locally through their separate variables, which is convenient for more clinicians to understand the significance of the prediction model. Survival analysis models typically output functions (survival or risk functions) rather than point predictions like regression and classification models. This makes interpreting these models a challenging task, especially with Shapley values. To do this, we apply SurvSHAP, a new model agnostic algorithm, to interpret survival models that predict survival curves. The algorithm is based on finding patterns in the predictive survival curve that will identify significantly different survival behaviors, and utilizing proxy models and SHAP methods to explain these different survival behaviors. Experiments on both synthetic and real datasets show that SurvSHAP is able to capture the underlying factors of survival patterns. In addition, the SurvSHAP results of the Cox proportional risk model are compared with the weights of the model to show that we provide a more realistic overall explanation and a more refined explanation of subpopulations. Non-linear machine learning survival models using SurvSHAP can better model the data and provide better interpretations compared to linear models.

## Conclusion

In this study, we obtained human mitochondrial regulatory genes based on MitoCarta database, and conducted initial screening and sample clustering based on unsupervised consensus cluster analysis and Boruta algorithm. We explored the correlation between these gene expressions and the immune landscape of ccRCC, and found that mitochondrial pathways are associated with multiple immune cell infiltrations in ccRCC and with drug susceptibility to multiple clinically targeted drugs. We used the iterative LASSO algorithm to screen genes for several times and constructed COX and RSF prediction models at the same time. Comparing the prediction performance of the two models, we found that the prediction model constructed by random forest algorithm was comprehensively superior to the prediction model constructed by COX regression algorithm. Our analysis results indicate that the mitochondrial pathway has obvious guiding value for the clinical therapy of ccRCC.

### Supplementary Information

Below is the link to the electronic supplementary material.

Supplementary file1 (TIF 22535 KB)

Supplementary file2 (TIF 93 KB)

Supplementary file3 (TIF 165 KB)

Supplementary file4 (TIF 32120 KB)

Supplementary file5 (TIF 50754 KB)

Supplementary file6 (XLSX 905 KB)

## Data Availability

The data used to support the findings of this study are available from the corresponding author upon request.

## Abbreviations

RCC: Renal clear cell carcinoma
ccRCC: Clear cell renal cell carcinoma
VHL: Von Hippel-Lindau
PI3: Phosphatidylinositol-3
TCGA: The Cancer Genome Atlas
KIRC: Kidney renal cell carcinoma
GDSC: Genomics of Drug Sensitivity in Cancer
GO: Gene Ontology
KEGG: Kyoto Encyclopedia of Genes and Genomes
NK cells: Natural killer cells
LASSO: Least absolute shrinkage and selection operator
MDSC: Marine-derived suppressor cells
AUC: Area under ROC curve
ROC: Receiver Operating Characteristic
PCA: Principal Component Analysis
TCA cycle: Tricarboxylic acid cycle
MeTIL: Methylation score
IC50: Half maximal inhibitory concentration
pRCC: Papillary renal cell carcinoma
VEGF: Vascular endothelial cell growth factor
PDGF: Platelet-derived growth factor
mTOR: Mammalian target protein of rapamycin
HIF: Hypoxia-inducing factor
PDK: Pyruvate dehydrogenase kinase
ETC: The electron transport chain
MDR: Multidrug resistance
PT: Petasin
