# Supplementary figures and images for: Potential value of mitochondrial regulatory pathways in the clinical application of clear cell renal cell carcinoma: a machine learning-based study
Source: J Cancer Res Clin Oncol. 2023 Sep 25;149(19):17015–26. doi: 10.1007/s00432-023-05393-8 (PMC10657316; doi:10.1007/s00432-023-05393-8)

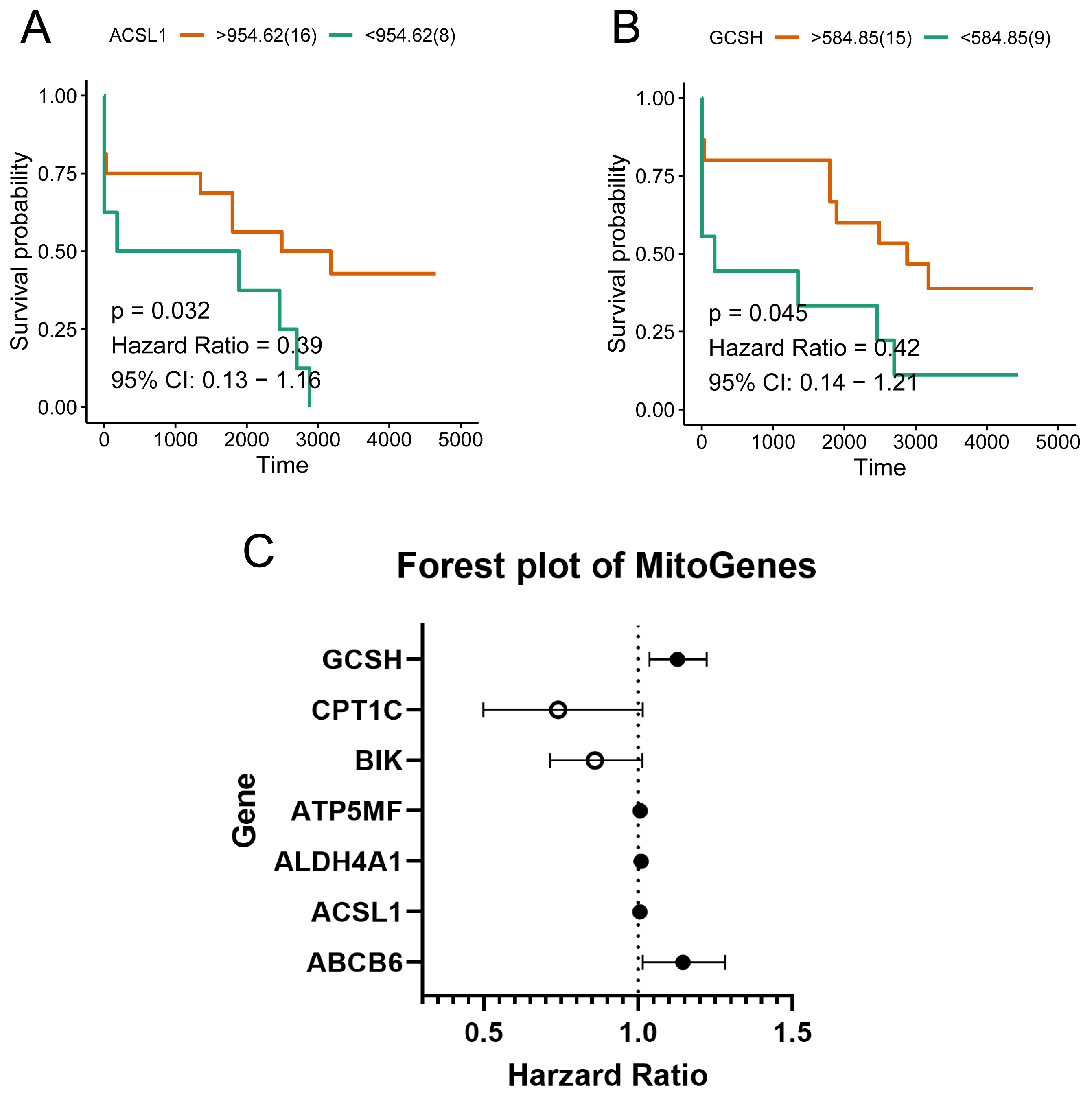

Supplement: Supplementary file 1 — Supplementary file1 (TIF 22535 KB) [file 432_2023_5393_MOESM1_ESM.tif]

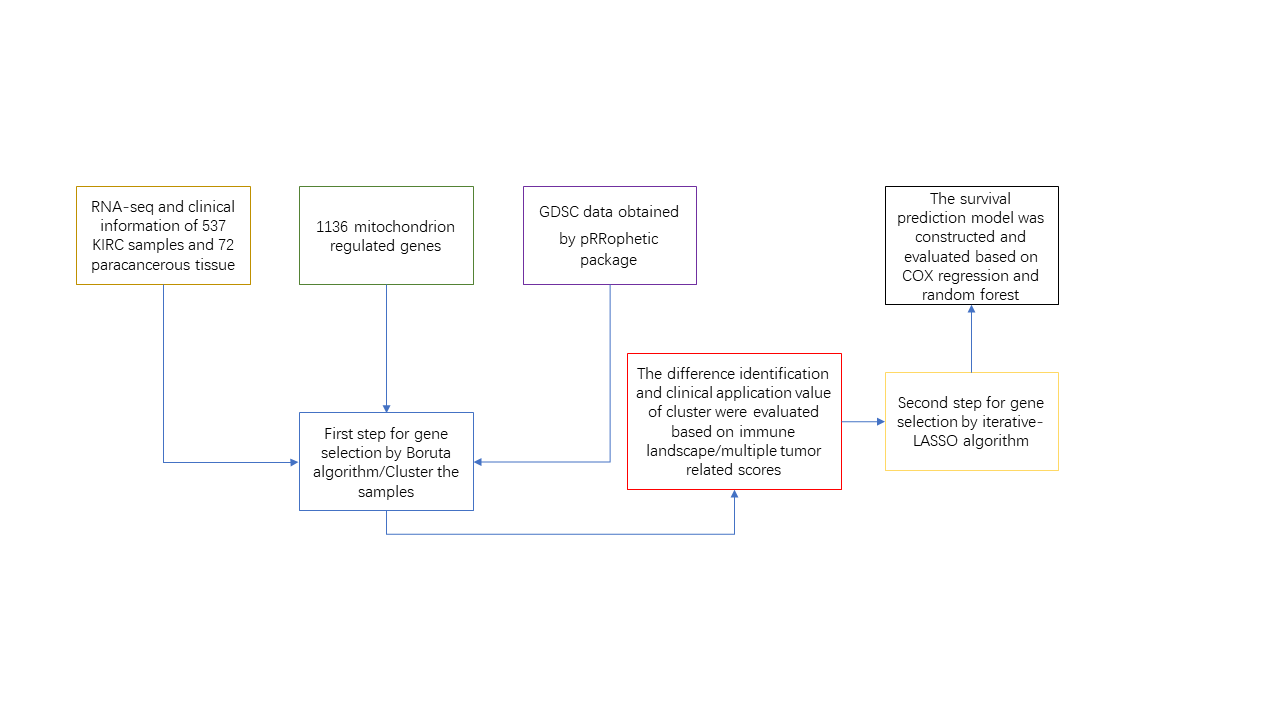

Supplement: Supplementary file 2 — Supplementary file2 (TIF 93 KB) [file 432_2023_5393_MOESM2_ESM.tif]

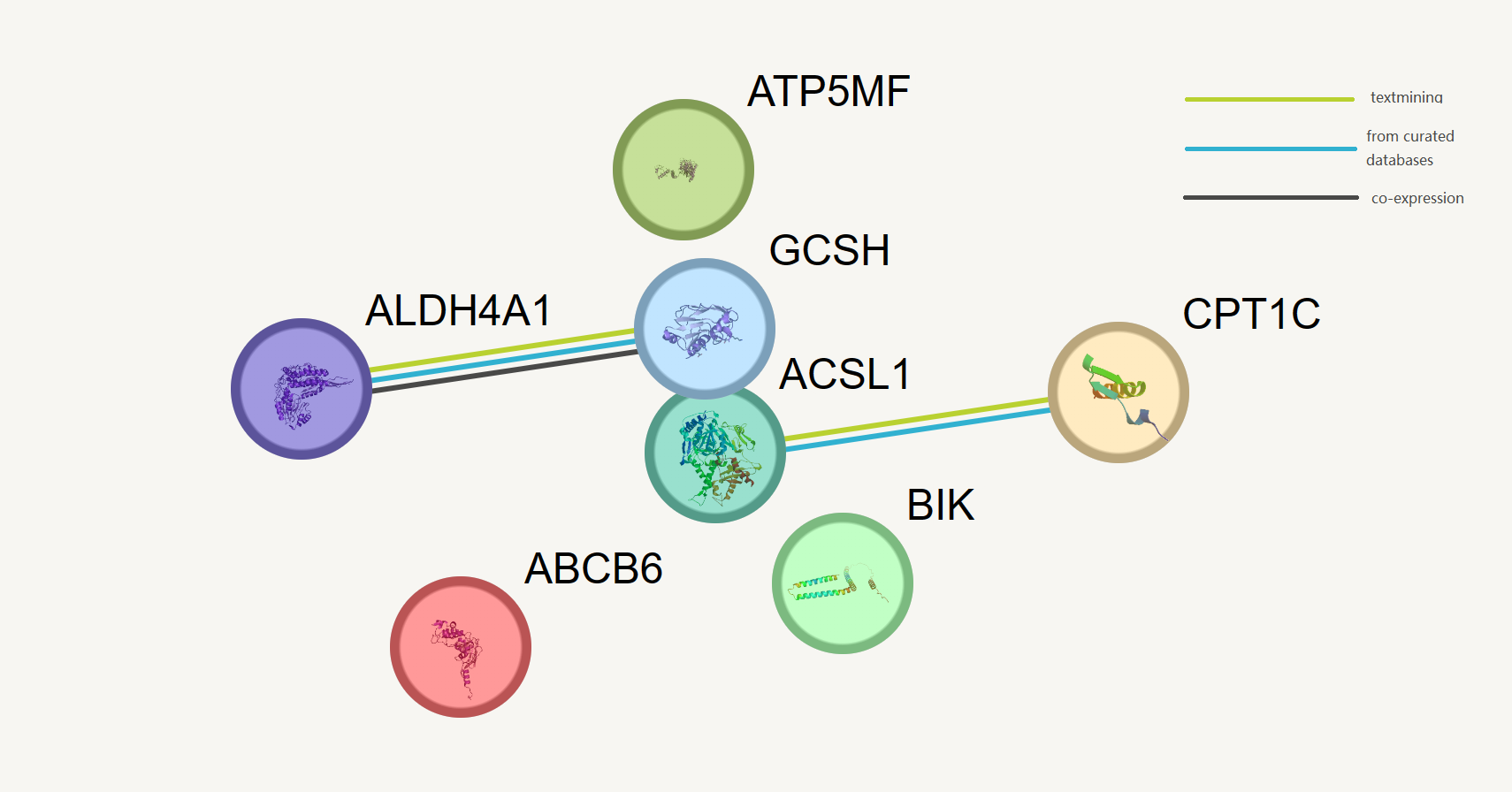

Supplement: Supplementary file 3 — Supplementary file3 (TIF 165 KB) [file 432_2023_5393_MOESM3_ESM.tif]

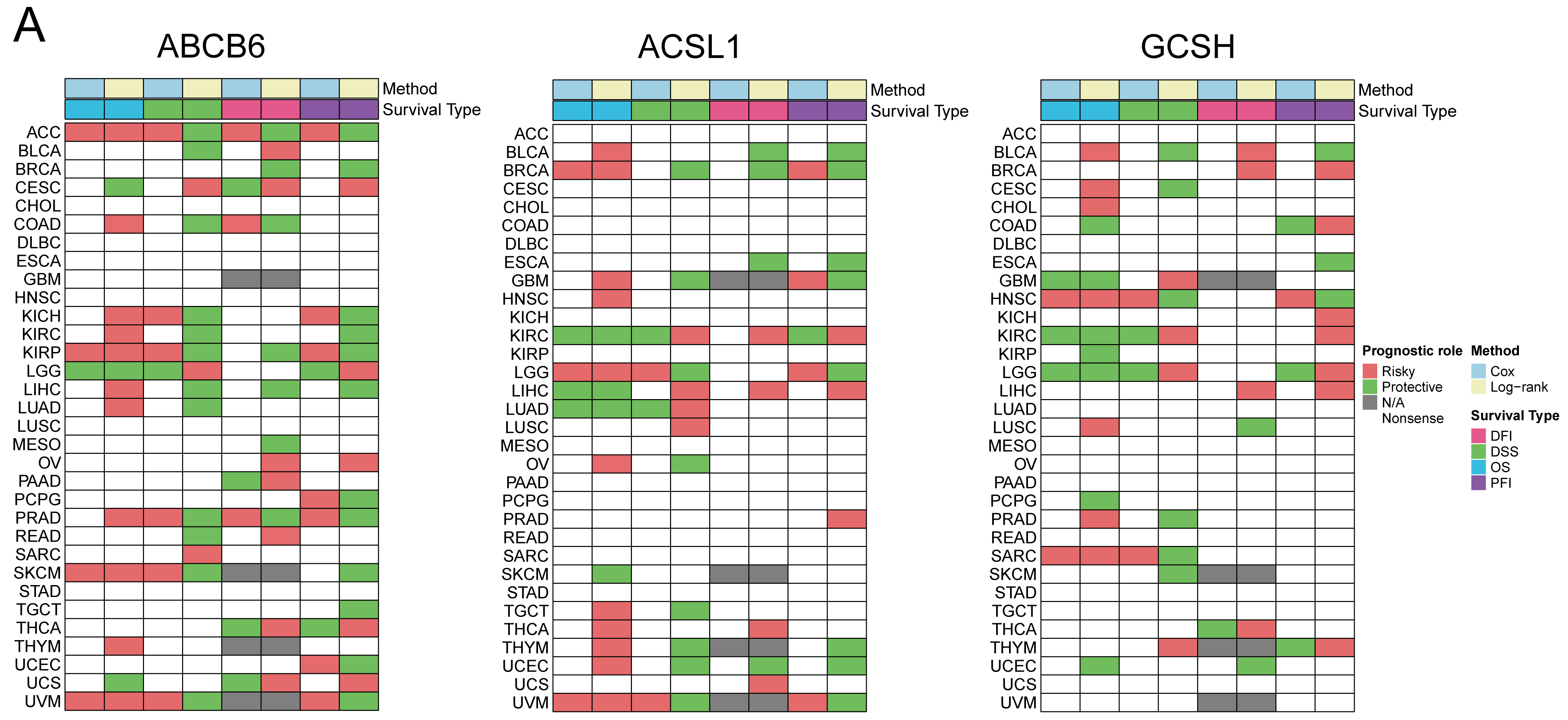

Supplement: Supplementary file 4 — Supplementary file4 (TIF 32120 KB) [file 432_2023_5393_MOESM4_ESM.tif]

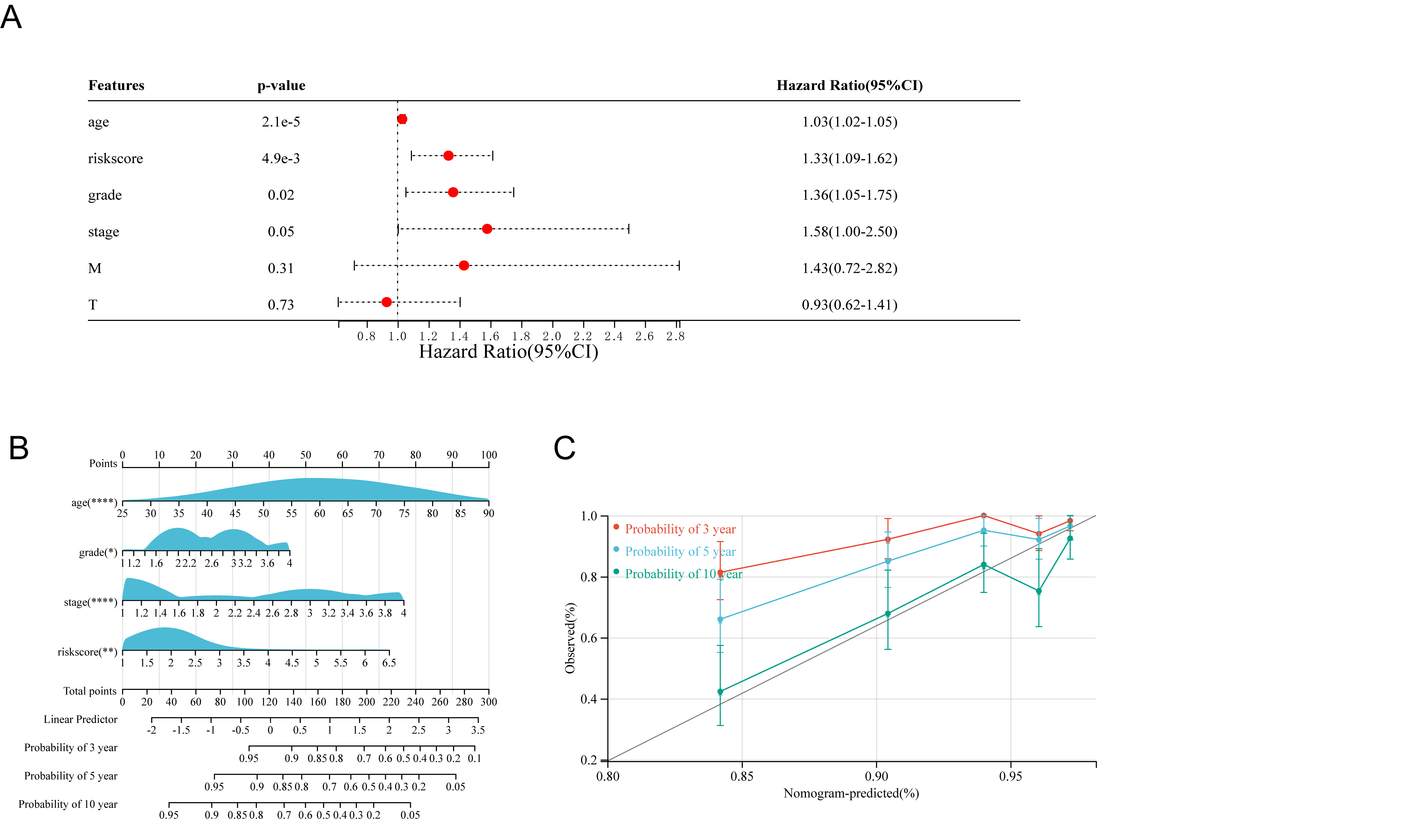

Supplement: Supplementary file 5 — Supplementary file5 (TIF 50754 KB) [file 432_2023_5393_MOESM5_ESM.tif]
